# Harmless Parasites

**Published:** 1901-11-16

**Authors:** 


### HARMLESS PARASITES.

The perpetually recurring question of the relation between the various factors which when acting together cause disease receives a somewhat new light from the suggestion lately thrown out by Mr. Jonathan Hutchinson, in the *Polyclinic*, in reference to the *rôle* of the normal, or, as he prefers to call them, the harmless parasites of the human body. He reminds us of the well-known fact that certain living organisms which habitually infest the human subject without producing any ill results may upon occasions manifest virulent properties. Of such parasites the pneumococcus and the bacillus coli communis are good examples. The pneumococcus is present in the nasal cavities from year to year in those who appear quite well, but it may at times cause pneumonia or fatal peritonitis. May we not then turn the doctrine of normal parasites round the other way, and ask whether some of the parasites which according to our present knowledge are always pathogenic may not under certain circumstances pass through long periods of innocency? If such be the case our next task must be to discover the kind of influences under which their aptitudes change and they become virulent. The bacillus of tubercle, for example, may it become under favourable conditions a harmless parasite? And the same in regard to leprosy. "Of the latter we know from unquestionable evidence that a man may have shown no evidences whatever of its presence for 12 or 20 years from the probable date of its reception"; while of tuberculosis we know that "after a temporary manifestation of activity it may, so to speak, go to sleep for 30 years and at the end of that time wake up into renewed activity in the self-same structure which was its first home." Can we then fairly say that during these long intervening periods the parasite has been present as a "normal" one? or are we to imagine that, by its activity years before, it has produced a local tissue weakness of so specific a nature and of so durable a character that on a fresh exposure to infection the parasite again sought and found its home in the same spot as had been affected by the disease 30 years beforehand? The latter surely is a difficult doctrine, and the suggestion made by Mr. Hutchinson, namely, that just as the pneumococcus, which is sometimes recognised as the virulent cause of a fatal disease, may as a general thing be a harmless or normal parasite of the body, so the bacillus tuberculosis, mostly known to us though it be as the cause of a fatal disease, may at other times exist as a harmless parasite. These are thoughts rather than fully formed theories. But they are full of suggestiveness.

